# Four years of daily stable water isotope data in stream water and precipitation from three Swiss catchments

**DOI:** 10.1038/s41597-022-01148-1

**Published:** 2022-02-10

**Authors:** Jana von Freyberg, Andrea Rücker, Massimiliano Zappa, Alessandro Schlumpf, Bjørn Studer, James W. Kirchner

**Affiliations:** 1 School of Architecture, Civil and Environmental Engineering, EPFL, 1015 Lausanne, Switzerland; 2 Mountain Hydrology and Mass Movements, Swiss Federal Institute for Forest, Snow and Landscape Research (WSL), 8903 Birmensdorf, Switzerland; 3 Department of Environmental Systems Science, ETHZ, 8092 Zurich, Switzerland; 4 Department of Earth and Planetary Science, University of California, Berkeley, CA 94720 USA

**Keywords:** Hydrology, Environmental monitoring, Hydrology

## Abstract

Time series of the natural isotopic composition (^2^H, ^18^O) of precipitation and streamwater can provide important insights into ecohydrological phenomena at the catchment scale. However, multi-year, high-frequency isotope datasets are generally scarce, limiting our ability to study highly dynamic short-term ecohydrological processes. Here we present four years of daily isotope measurements in streamwater and precipitation at the Alp catchment (area 47 km^2^) in Central Switzerland and two of its tributaries (0.7 km^2^ and 1.6 km^2^). This data set reveals short-term responses of streamflow isotopes to precipitation events, which otherwise remain obscured when isotopes are sampled weekly or monthly. The observations span the period June 2015 through May 2019, during which several hydrometeorologic extreme events occurred, including a very dry summer in 2018 and below-average snow accumulation in winter 2016/2017. In addition, we provide daily time series of key hydrometeorological variables that, in combination with the isotope data, can be useful for assessing the robustness of ecohydrological models.

## Abstract (summary)

**Table:** 

Measurement(s)	((18)O)water • deuterium(.) • waterflux • Relative Humidity • Air temperature • Snow depth
Technology Type(s)	wavelength scanned cavity ring-down spectrometer • off-axis integrated cavity output spectrometry • stream gauge, rain gauge • weather station
Factor Type(s)	catchment • location_ID • source • waterflux_measured • precipitation_interpolated • analysis_method • delta_2H • delta_18O • rel_humidity • air_temperature • snow_depth • notes_sampling • notes_other
Sample Characteristic - Environment	continental temperate

Machine-accessible metadata file describing the reported data: 10.6084/m9.figshare.17013848

## Background & Summary

Stable isotopes of water (particularly ^2^H and ^18^O) are widely used as natural tracers to study hydrological and ecological processes^1,2^. For example, temporal variations of isotope values in streamflow and precipitation can be compared to estimate the relative contributions of recent precipitation to streamwater or evapotranspiration^3,4^. Time series of stable water isotopes can also be used to quantify how quickly rainwater becomes streamflow^5–7^ and estimate catchment water storage^8^. The isotopes ^2^H and ^18^O are considered to be ideal conservative tracers because their concentrations in streamwater are mainly affected by mixing of different waters. Other processes that could substantially alter these isotope values, such as evaporative isotopic fractionation, are usually negligible once incoming precipitation has infiltrated into the subsurface^9^.

Until recently, most ecohydrological studies have relied on chemical and isotopic tracers sampled at weekly or even monthly intervals (e.g.^4,8,10^, although streamflow often responds to precipitation orders of magnitude faster than this, particularly in small catchments^11^. This mismatch in timescales has made it difficult to use these tracers to illuminate the hydrological and ecological functioning of landscapes^12,13^. A further impediment has been the scarcity of tracer data from nested catchments spanning a range of spatial scales.

Precipitation and streamwater isotope time series have typically been compiled in individual catchment-specific research projects and have rarely been made public. The scarcity of publicly available data impedes cross-catchment comparison studies and multi-site applications of tracer-aided models, both of which are essential to scale up our mechanistic understanding of hydrological processes beyond individual study sites. In Europe, few multi-year isotope datasets have been published so far. Several years of isotope measurements are available at weekly frequency for 23 Swiss catchments^14^, at 7-hourly and weekly frequency at Plynlimon, Wales^5^, at daily frequency at the Scottish Bruntland Burn catchment^15^, at weekly frequency at the German Wüstebach catchment^16^, and at daily and weekly frequency for the Italian Ressi catchment^17^.

This paper complements these existing datasets by presenting four-year (June 2015–May 2019) time series of daily δ ^2^H and δ^18^O values in streamwater and precipitation from the Swiss Alptal research catchment. Streamwater isotopes were measured in the Alp main stream and in two of its tributaries (Erlenbach and Vogelbach); precipitation isotopes were measured at two grassland locations in the Alptal catchment, in the headwaters at 1228 m a.s.l. and near the outlet at 910 m a.s.l. The dataset also includes daily time series of key hydrologic and meteorologic variables, such as daily streamwater and precipitation fluxes, air temperature, relative humidity and snow depth. By including these additional time series, we hope to facilitate a straightforward application of the dataset by researchers worldwide.

## Methods

### Catchment properties

**Fig. 1:**
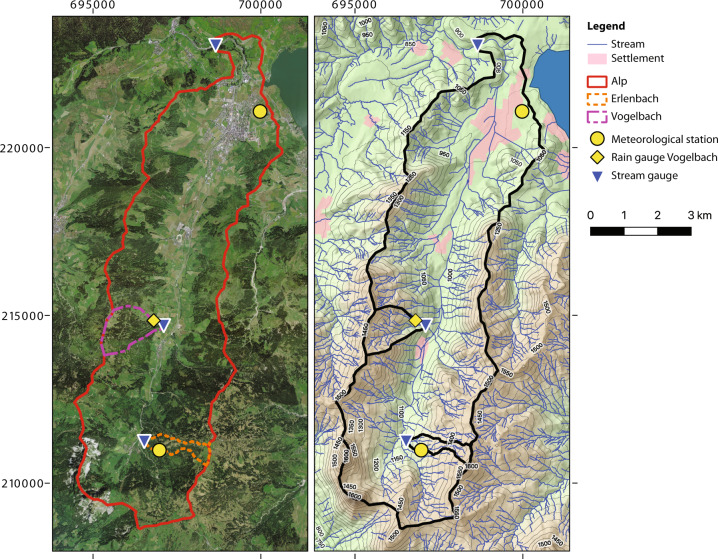
Sampling and measurement locations in the Alp catchment and in two of its tributaries, the Erlenbach and the Vogelbach catchments. Isotope samples were collected at the meteorological station and the stream gauges, but not at the Vogelbach rain gauge. The coordinate system is CH1903/LV03.

The 46.4 km^2^ Alp catchment is located near the city of Einsiedeln in central Switzerland (Fig. 1). The catchment spans an elevation range of 1058 m, with the outlet at 840 m a.s.l. (m above sea level) and the highest summit (Grosser Mythen) at 1898 m a.s.l. The average slope of the Alp catchment is 16° with a flat valley bottom and very steep slopes of up to 75° at the south-western catchment boundary. Isotopes were also sampled in two smaller tributaries of the Alp river: the 0.7 km^2^ Erlenbach catchment on the eastern side of the Alptal valley (elevation range from 1080 to 1520 m a.s.l.), and the 1.6 km^2^ Vogelbach catchment on the western side of the Alptal valley (elevation range from 940 to 1480 m a.s.l.). Table 1 provides an overview of the sampling and measurement locations.

**Table 1:** Properties and measured variables at the sampling locations in the Alp catchment.

Catchment	Site description	Longitude (WGS84)	Latitude (WGS84)	Elevation (m above sea level)	Measured variables	Data access
Alp	Streamflow gauge	8.73928E	47.15080 N	840	River discharge	Swiss Federal Office of the Environment (FOEN)
					δ^2^H, δ^18^O in streamwater	This dataset
Alp	Meteorological station	8.75708E	47.13370 N	910	Precipitation amount	MeteoSwiss
					δ^2^H, δ^18^O in precipitation	This dataset
Erlenbach	Streamflow gauge	8.70921E	47.04480 N	1180	River discharge	This dataset
					δ^2^H, δ^18^O in streamwater	This dataset
Erlenbach	Meteorological station	8.71502E	47.04249 N	1228	Precipitation amount	This dataset
					δ^2^H, δ^18^O in precipitation	This dataset
Vogelbach	Streamflow gauge	8.71614E	47.07621 N	1050	River discharge	This dataset
					δ^2^H, δ^18^O in streamwater	This dataset
Vogelbach	Rain gauge	8.71336E	47.07758 N	1145	Precipitation amount	This dataset

The bedrock geology of the Alp catchment consists of tertiary flysch (sandstone, limestone, clays and marls) and subalpine molasse (conglomerates, sandstone, and marls); the valley bottoms are overlain by gravel and landslide material from the adjacent hillslopes. Soils are generally shallow, with low permeability. The flanks of the Alp catchment are dominated by forests, grasslands and wetlands, and the valley bottom is dominated by summer pastures and settlements^18^ (Burch *et al*., 1996). More information about these catchment properties can be found on the online map service of the Swiss Federal Office of Topography swisstopo (URL: https://map.geo.admin.ch, accessed 17 July 2021).

Total annual precipitation in the Alp valley is strongly controlled by elevation, averaging 1791 mm y^−1^ in the flat northern part near the outlet, and roughly 30% more in the mountainous headwaters of the catchment (2300 mm y^−1^^18,19^). Snowfall comprises up to one-third of total precipitation in the headwaters of the Alp^19,20^, although snowfall is frequently interrupted by rainfall during mild periods in winter^21,22^. Mean monthly air temperatures show a distinct seasonal pattern, ranging from −1.9 °C in February to 15.9 °C in July at the Erlenbach meteorological station in the headwaters, and ranging from −1.2 °C in February to 17.7 °C in July at the Einsiedeln meteorological station near the outlet. A detailed description of the long-term hydrometeorological measurements in the Alptal catchment is provided by Stähli *et al*.^23^.

### Precipitation sampling

Between June 2015 and September 2017, daily composite samples of precipitation (rain and snowmelt) were collected at the Einsiedeln and Erlenbach meteorological stations (denoted EIN_meteo and ERL_meteo, respectively) with unheated precipitation collectors (Palmex d.o.o., Zagreb, Croatia). In the rainy season (around 1 June–31 October), we used a 13.5-cm diameter plastic funnel. This was replaced by a 30-cm long extended aluminum funnel with 15 cm diameter in the snowy season (around 1 November–31 May). The orifices of the 13.5-cm and 15-cm diameter funnels were located at 2 m and 2.3 m above ground, respectively. We did not use the Palmex sampling bottle and pressure-compensation tube, but instead attached an automatic water sampler (6712-Fullsize Portable Sampler, Teledyne Isco, Lincoln, Nebraska, USA) with a silicone tube to the funnel outlet. This allowed the precipitation sample to drain by gravity into one of 24 dry HDPE sample bottles inside the automatic water sampler. Every day at 5:40 AM, the injection arm of the automatic water sampler rotated to the next empty sample bottle. Because each automatic sampler can hold up to 24 bottles, the filled sample bottles were retrieved and replaced with dry bottles after up to 24 days.

Each sample bottle was equipped with an evaporation protection system, consisting of a syringe housing with attached silicone tube that is inserted into the opening of the sampler bottles. Because the silicone tube reaches beneath the surface of the water sample in the bottle, the contact area between the water sample and the atmosphere outside the bottle is minimized. Extensive laboratory and field experiments with this evaporation protection system verified that isotope fractionation effects in the collected water samples (due to evaporation and vapor mixing) are negligible over sample storage durations of up to 24 days^24^. We nonetheless monitored for any potential fractionation effects using control samples of known isotopic composition kept in identical sample bottles within the autosampler during each sampling period.

In the snowy season, a heating cable was attached to the silicon tube to prevent freezing of the water sample. The extended aluminum funnel used for winter sample collection was painted black to accelerate the melting of the captured snow, thus reducing evaporative fractionation effects of the meltwater sample in the funnel^25,26^. Rücker *et al*.^27^ evaluated this unheated precipitation collector with the black-colored funnel during two snowmelt periods in April and November 2016, and showed that the precipitation volumes can be significantly affected by under-catch; however, the isotopic composition of the precipitation (rain and naturally melted snow) was similar to the isotopic composition of the snowpack outflow^27^.

On 5 September 2017, the unheated precipitation collectors were replaced by heated precipitation collectors (52202 Electrically Heated Rain and Snow Gage, Campbell Scientific, Loughborough, UK) at both meteorological stations. Because the factory-supplied heating of the Campbell rain gage did not prevent freezing of the tipping bucket mechanism during very cold conditions, we painted the outer housing black and installed an additional resistor (100 Ω, 25 W) beneath the tipping bucket. The resistor and the factory-supplied heating pad were automatically activated as soon as air temperature fell below 4 °C.

Because the unheated precipitation collectors were replaced with heated ones only in September 2017, some precipitation isotope values during the winters 2015/2016 and 2016/2017 represent naturally-melted snow and ice that had been collected earlier in the unheated funnel during freezing conditions. Therefore, precipitation isotope values may lag their associated precipitation volumes, which were always measured with heated rain gauges, by up to several days during these periods (e.g., in January and February 2017 at the Erlenbach meteorological station). In the winters of 2015/2016 and 2016/2017, precipitation samples that fell during freezing conditions but did not melt in the funnel on the same day are reflected in the data set by days with recorded precipitation (from the heated rain gauges) but no isotope values. These missing isotope values could potentially be in-filled from isotope values on subsequent days (when the frozen samples presumably melted), but we have not done so here. Instead, we have used measurements of air temperature and precipitation amounts to estimate whether a sampling day might have been affected by accumulation or melt of snow in the unheated collection funnel at the Erlenbach and Einsiedeln meteorological stations. Days were flagged as “snow accumulation” when measured precipitation was ≥1 mm d^−1^, air temperature was <4 °C and snow depth increased the following day; analogously, days were flagged as “snowmelt” when no precipitation was measured, air temperature was ≥4 °C and snow depth decreased the following day.

To allow an individual assessment of the precipitation isotope data quality, we provide additional information on sample amounts and *lc*-excess values^28^. The sample volumes were determined by weighting the filled sample bottles and subtracting their empty weights. We classified samples of 10 ml or less as not reliable because these samples were associated with the most negative *lc*-excess values, i.e. they were evaporatively fractionated. Whereas other, larger precipitation samples have been collected for which the *lc*-excess values were negative as well, we cannot say with certainty whether these samples reflect the true precipitation isotope signal or whether these samples have been affected by fractionation or vapor mixing after collection.

### Streamwater sampling

Composite stream water samples were collected with automatic water samplers (6712-Fullsize Portable Sampler, Teledyne Isco, Lincoln, Nebraska, USA) at the outlets of the Erlenbach, Vogelbach and Alp catchments. Four times per day, at 5:40 AM, 11:40 AM, 5:40 PM, and 11:40 PM (UTC + 1), the samplers pumped 100 ml of stream water into a dry 1-litre HDPE bottle. This sampling schedule was used because MeteoSwiss meteorological measurements are generally aggregated to daily values for the time period 5:40 AM - 5:39 AM of the following day. Note that our streamwater sampling strategy (i.e., four composite grab samples) is different from that for precipitation (i.e., cumulative integrated sampling from 5:40 AM till 5:39 AM of the following day; see Sect. 2.2). Note also that precipitation falling in the last six hours (11:40 PM to 5:40 AM) of each precipitation sampling day will first be reflected in the streamwater samples of the following day.

Because each automatic sampler can hold up to 24 bottles, the filled sample bottles were retrieved and replaced with dry bottles after up to 24 days. To avoid isotopic fractionation effects in the collected water samples during that storage period, we retrofitted the Teledyne Isco sampler bottles with the evaporation protection system described in detail in von Freyberg *et al*.^24^.

The automatic streamwater samplers were installed inside huts (Erlenbach, Vogelbach) and in an underground room (Alp) in which air temperatures were kept above freezing. In the hut at the Erlenbach catchment outlet, we installed the streamwater sampling tube along a vertical shaft that was located inside the hut and provided direct access to a bypass channel of the Erlenbach stream. At the outlet of the Vogelbach catchment, no shaft existed so the sampling tube was laid from the sampler inside the hut into the stream. The sampler inside the underground room at the Alp catchment outlet was connected to the stream via a 17-m long tube inside an underground pipe. The setups at the Alp and Vogelbach catchments resulted in occasional sample loss in winter when streamwater froze inside the sampling tube. In addition, sampling at the Vogelbach stream was interrupted several times between August and November 2017 due to rodent damage of the sampling tube. At the Alptal catchment outlet, the ISCO automatic water sampler broke and was replaced by a different autosampler (Maxx P6L – Vacuum System, Maxx GmbH, Rangendingen, Germany) on 21 December 2018. No evaporation protection was used in the Maxx P6L sampler because its bottles have different dimensions than those in the 6712 Teledyne Isco samplers., and because the Maxx P6L sampler was located in an underground monitoring station where temperature and humidity fluctuations were much smaller than outdoors. We verified that any fractionation was less than twice our analytical uncertainty using control samples of known isotopic composition kept in open sample bottles within the Maxx P6L autosampler throughout each sampling period.

### Sample handling and isotope analysis

All samples were stored in sealed autosampler bottles at 4 °C until sample filtration (any frozen water samples were melted at room temperature before storage). The samples were filtered through 0.45-μm Teflon filters (DigiFilter micron Teflon, S-Prep GmbH, Überlingen, Germany; WIC 80345, WICOM, Heppenheim, WICOM Germany GmbH) and 1.5 ml of filtrate was transferred into autosampler glass vials. Whenever possible, the water samples were analyzed in batches with each batch comprising all samples collected within a 24-day sampling period. This means that each analysis batch comprised up to 72 (3 × 24) streamwater samples and up to 48 (2 × 24) precipitation samples.

Water samples collected between 1 June and 30 July 2015 were measured at the central laboratory of the Swiss Federal Institute for Forest, Snow and Landscape Research (WSL) using a wavelength-scanned cavity ring-down spectrometer CRDS (L2130-*i*, Picarro Inc., Santa Clara, California, USA). Water samples collected between 31 July 2015 and November 2017 were analyzed at WSL using an off-axis integrated cavity output spectrometer OA-ICOS (Triple Isotope Water Analyzer TIWA-45EP, ABB Los Gatos Research, San Jose, California, USA). Both instruments were calibrated by measuring five commercially available reference standards (LGR1-LGR5) every 20 to 25 samples of each batch. For each batch, the average values of these standard isotope measurements were used to obtain the linear calibration equation and to determine instrument drift. For additional validation, two quality control standards (commercial standard Medium Natural Water B2193 WA101B, Elemental Microanalysis Ltd; internal standard “Sion water”) were measured every 20 to 25 samples. The long-term, post-calibration analytical precisions of both analyzers, i.e. 1 *σ* of repeated measurements of VSMOW2, were on average better than 0.5‰ for δ^18^O and 1‰ for δ^2^H.

Water samples collected after November 2017 were measured at the laboratory of the Physics of Environmental Systems group at ETH Zurich with a wavelength-scanned CRDS (L2130-*i*, Picarro Inc., Santa Clara, California, USA). Before the measurement of each batch, the instrument was calibrated with five commercially available working standards (LGR1-LGR5, Table 2). In addition, quality control standards (ENAN, CSIB, STW, and Fiji, Table 2) were measured after every 20 samples to quantify instrument drift and to validate the isotope measurements. We corrected for instrument drift using the slope and intercept of a linear regression fit between the line numbers and the post-calibration ENAN isotope values. The long-term average, post-calibration analytical precision for the instrument was generally better than 0.2‰ for δ^18^O and 1‰ for δ^2^H, based on repeated measurements of ENAN. A small number (35) of streamwater samples from the Vogelbach catchment were analyzed at ETHZ with an OA-ICOS (Triple Isotope Water Analyzer TIWA-45EP, ABB Los Gatos Research, San Jose, California, USA). Instrument calibration and measurement validation were carried out analogously to those of the CRDS at ETHZ. We ensured consistency between the different instruments at WSL and ETHZ through using the same calibration standards (LGR1-LGR5) that were regularly referenced to the international IAEA standards VSMOW2 and SLAP.

**Table 2:** Isotope values and standard deviations of the calibration and quality control standards used at the WSL and ETHZ laboratories.

Standard	Instruments	δ^18^O (‰-VSMOW)	δ^2^H (‰-VSMOW)
VSMOW2	LGR_WSL, PIC_WSL	0.00 ± 0.02	0.0 ± 0.3
B2193	LGR_WSL, PIC_WSL	−12.34 ± 0.13	−98.32 ± 1.13
Sion water	LGR_WSL, PIC_WSL	—	13.387
LGR1	LGR_WSL, PIC_ETHZ	−19.49 ± 0.15	−154.0 ± 0.5
LGR2	LGR_WSL, PIC_ETHZ	−16.24 ± 0.15	−123.7 ± 0.5
LGR3	LGR_WSL, PIC_ETHZ	−13.39 ± 0.15	−97.3 ± 0.5
LGR4	LGR_WSL, PIC_ETHZ	−7.94 ± 0.15	−51.6 ± 0.5
LGR5	LGR_WSL, PIC_ETHZ	−2.69 ± 0.15	−9.2 ± 0.5
ENAN	LGR_ETHZ, PIC_ETHZ	−11.74	−82.05
CSIB	LGR_ETHZ, PIC_ETHZ	−12.21	−88.3
STW	LGR_ETHZ, PIC_ETHZ	−14.13	−93.31
Fiji	LGR_ETHZ, PIC_ETHZ	−7.13	43.59

All instruments analyzed six injections per vial. We report the average and standard deviation of the last three injections; the first three injections were discarded as they are likely to be affected by memory effects from the previous sample. If the standard deviation was much larger than the analytical uncertainty of the instrument, either the two last injections were averaged (if the fourth injection differed markedly from the fifth and sixth) or the sample was measured again.

The isotopic abundances of ^18^O and ^2^H are reported using the *δ* notation relative to the IAEA standard Vienna Standard Ocean Water (VSMOW), after^9^:

1 $$\delta =\left(\frac{{R}_{{\rm{sample}}}}{{R}_{{\rm{VSMOW}}}}-1\right)\cdot 1000\textperthousand .$$

In Eq. (1), *R* is the ratio of the heavier isotope relative to the lighter isotope (i.e., ^18^O/^16^O or ^2^H/^1^H).

### River discharge and precipitation water fluxes

In our dataset we provide daily averages of river discharge at the outlets of the Erlenbach and Vogelbach catchments, daily precipitation amounts as measured at the Erlenbach meteorological station and the Vogelbach rain gauge (Table 1). In addition, we provide interpolated basin-average daily precipitation amounts for the Alp, Erlenbach and Vogelbach catchments, and daily measurements of air temperature, relative humidity and snow depth from the Erlenbach meteorological station.

Alp river discharge is measured by the Swiss Federal Office of the Environment (FOEN; URL: https://www.hydrodaten.admin.ch/de/2609.html, accessed 17 July 2021) at 10-minute intervals using a concrete rectangular flume. These data cannot be provided with our dataset due to legal restrictions; however, they can be requested free of charge from FOEN’s hydrology department (URL: https://www.bafu.admin.ch/bafu/en/home/topics/water/state/data/obtaining-monitoring-data-on-the-topic-of-water/hydrological-data-service-for-watercourses-and-lakes.html, accessed 17 July 2021).

The meteorological station near the city of Einsiedeln is operated by the Swiss Federal Office of Meteorology and Climatology (MeteoSwiss). The station provides measurements of precipitation rates, air temperature and relative humidity at 10-minute resolution, as well as a snow depth measurement at 6:00 am each day. Due to legal restrictions we cannot provide these data as part of our dataset, but they are freely available for research and teaching purposes from the Swiss Federal Office of Meteorology and Climatology (MeteoSwiss), Operation Centre 1, P.O. Box, CH-8058 Zurich Airport or from their data archive IDAWEB (URL: https://gate.meteoswiss.ch/idaweb/login.do, accessed 17 July 2021).

Hydrological data from the Erlenbach and Vogelbach catchment outlets and meteorological data from the Erlenbach meteorological station can be requested directly from WSL’s mountain hydrology and mass movements research unit (URL: https://www.wsl.ch/en/about-wsl/instrumented-field-sites-and-laboratories/experimented-field-sites-for-natural-hazards/torrent-investigation-in-the-alptal/data.html, accessed 17 July 2021). The design of the Vogelbach stream gauge does not allow for reliable low-flow measurements. For instance, during the summer drought 2018, the stream never dried out although zero flows were recorded occasionally in July and August. To correct for this, we interpolated unrealistic Vogelbach discharge data using discharge measurements from the Alp river. All of the rain gauges were heated; thus we cannot distinguish between snowfall and rainfall.

In our dataset, we provide daily, basin-average precipitation amounts estimated using the WINMET pre-processing tool of the rainfall-runoff model PREVAH^29^. The WINMET tool used daily precipitation data (aggregated between 5:40 AM and 5:39 AM of the next day) from all available rain gauges in the area (i.e., 30 MeteoSwiss stations and additional rain gauges in the Alp, Erlenbach and Vogelbach catchments; Table 2) and digital terrain models of the three study catchments to estimate daily precipitation volumes for 100-meter altitude bands. Based on the areal proportions of each 100-meter elevation band in each catchment, the area-weighted basin average precipitation volumes could be calculated. We did not explicitly calculate snowfall versus rainfall because all rain gauges were heated.

## Data Records

Precipitation isotope values at the two meteorological stations range from −25.39 to −0.01 for δ^18^O and from −194.55 to 4.65 for δ^2^H. In contrast, isotopes in streamwater are less variable, ranging from −14.47 to −6.39‰ for δ^18^O and from −102.97 to −43.05‰ for δ^2^H. Because the water vapor sources of precipitation in the Alptal catchment are diverse^30^ and precipitation events are frequent^19^, precipitation isotope values vary substantially from day to day. These precipitation events, and snowmelt events (occurring predominantly between April and June), are both reflected in short-term variations in streamwater isotopes.

Positive isotope values (and/or negative *lc*-excess values) were occasionally observed in precipitation, usually associated with small precipitation volumes and high air temperatures. These high values may have resulted from fractionation of the raindrops during their fall through a warm dry atmosphere^31,32^, or potentially from evaporative fractionation from the surface of the rain funnel. Our control samples (see Sections 2.2 and 2.3) suggest that evaporative fractionation within the autosamplers is unlikely to be the reason for these positive isotope values.

**Fig. 2:**
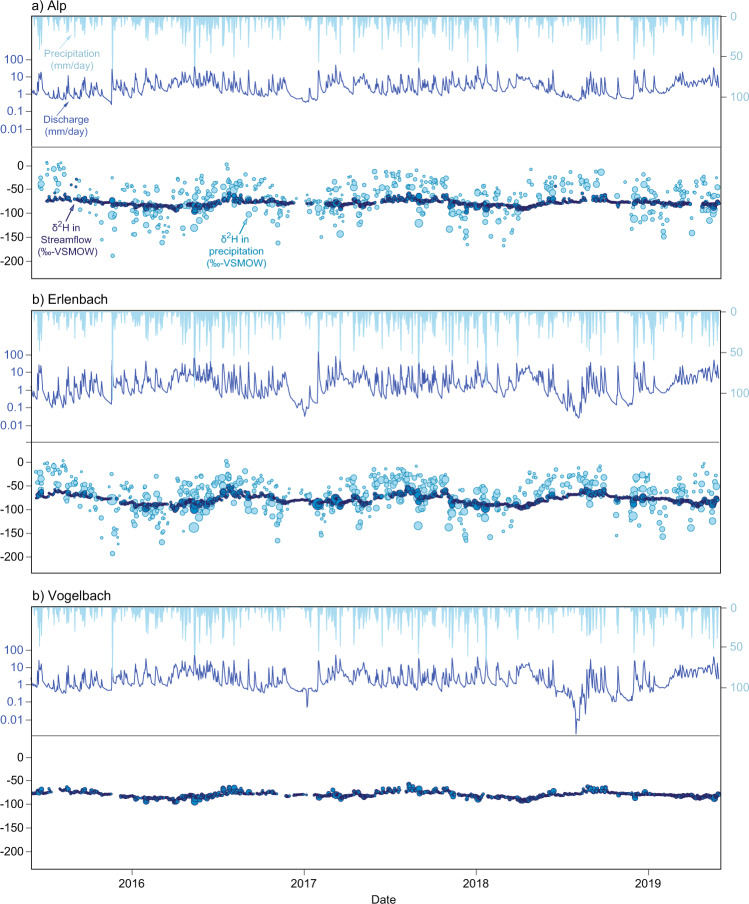
Time series of deuterium (δ^2^H) in streamwater (dark blue) and precipitation (light blue), as well as daily river discharge and precipitation amounts in the catchments of the (**a**) Alp, (**b**) Erlenbach, and (**c**) Vogelbach. No precipitation samples were collected in the Vogelbach catchment. The size of the deuterium data points corresponds to the measured water flux.

Precipitation and streamwater isotopes follow a distinct seasonal pattern with heavier isotopes in summer and lighter isotopes in winter (Fig. 2). The seasonal streamwater isotope amplitude is damped compared to that of precipitation due to mixing with older water in catchment storage^33^.

**Fig. 3:**
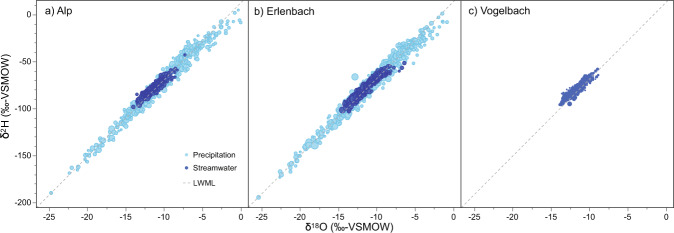
Dual-isotope plots of precipitation (light blue) and streamwater (dark blue) isotope values measured at the outlets and meteorological stations of the (**a**) Alp catchment, (**b**) Erlenbach catchment, and (**c**) Vogelbach catchment (only streamwater isotopes). The size of the data points corresponds to the measured water flux. The LWML was estimated from monthly, amount-weighted precipitation isotope data at both the Alp and Erlenbach meteorological stations. Only precipitation isotope data with quality levels 1 and 2 were used in this figure and to determine the LMWL.

The local meteoric water line (LMWL), based on monthly volume-weighted δ^2^H and δ^18^O values of precipitation at both the Alp and Erlenbach meteorological stations, was determined by precipitation weighted least-squares regression. This regression approach was used to reduce the effect of extreme values of small precipitation events^34^. The resulting LMWL(±standard error) is δ^2^H = 12.9(±1.5) + 8.2(±0.1)∙δ^18^O (R^2^ = 0.98, p < 0.0001, n = 94; Fig. 3), which closely follows the Global Meteoric Water Line (GMWL, δ^2^H = 10 + 8∙δ^18^O). A linear regression based on daily isotope and basin-average precipitation data is very similar, i.e. δ^2^H = 11.3(±0.5) + 8.0(±0.05)∙δ^18^O (R^2^ = 0.97, p < 0.0001; n = 1029 Fig. 3).

Our calculation of the LMWL neglected 120 of 1260 precipitation isotope measurements that were associated with days on which the basin-average precipitation amounts were zero. The large majority of these days occurred during the snowfall-snowmelt periods between October 2015 and June 2017, when unheated precipitation collectors were used. When temperatures were near or below freezing, snow accumulated in the unheated collection funnels and once air temperatures increased, the accumulated snow would melt and contribute to the day’s precipitation sample, whereas this delayed snowmelt would not be accounted for in the heated tipping bucket measurements.

On the other hand, there were in total 337 days (around 15% of the total measured precipitation amount at both stations), during which precipitation volumes were in theory large enough to yield a precipitation sample but no samples were collected or the collected sample volume was below 10 ml. This calculation assumes a critical precipitation amount of 10 ml plus 0.2 mm wetting error, resulting in 138 days when precipitation was ≥0.56 mm day^−1^ during the rainy season, and 199 days with precipitation ≥0.5 mm day^−1^ during the snowy season. These no-sampling days account for 21% and 11% of the total precipitation amounts measured at the Einsiedeln and Erlenbach meteorological stations, respectively. Missing samples can be explained by the delayed melt of snow that had previously accumulated in the unheated precipitation collectors (until September 2017), as well as by power outages or other technical problems with the automatic samplers.

The dataset contains the daily streamwater and precipitation isotope data, as well as associated daily volumes of river discharge and precipitation from the Alp, Erlenbach and Vogelbach catchments and is archived in the EnviDat environmental data portal^35^ in a single table with 8766 rows and 20 columns^36^. The metadata of the data columns are provided in the Supplementary File “Data set Documentation” and in Table 3.

**Table 3:** Explanation of the data set.

Column Header	Explanation
date	The date that the sample was collected or the hydrometeorologic measurements were made. Stream samples consist of four combined 100-ml grab samples collected at 5:40 AM, 11:40 AM, 5:40 PM, and 11:40 PM (UTC+1) on the ‘date’. For precipitation samples, ‘date’ corresponds to the start of the sampling interval, which is from 5:40 AM of the current ‘date’ till 5:39 AM of the following one. Ten-minute measurements of relative humidity, temperature and snow depth were averaged to daily values over the interval between 5:40 AM of the current ‘date’ and 5:30 AM of the following one. Format: yyyy-mm-dd
catchment	Name of the study catchment
location_ID	Identifier of the sampling and/or measuring location
source	Distinguishes between precipitation or streamwater samples
waterflux_measured	Daily stream discharge (mm day^−1^) or precipitation fluxes (mm day^−1^) of the time interval between 5:40 AM of the current ‘date’ and 5:39 AM of the following one.
precipitation_interpolated	Daily, basin-average precipitation fluxes (mm day^−1^) of the time interval between 5:40 AM of the current ‘date’ and 5:39 AM of the following one.
analysis_method	Method and laboratory location of the water sample analyses: L2130-*i* Picarro at WSL and ETHZ (PIC_WSL, PIC_ETHZ), Triple Isotope Water Analyzer TIWA-45EP at WSL and ETHZ (LGR_WSL, LGR_ETHZ)
sample_volume	Volume of the collected precipitation sample (ml)
delta_2H	Deuterium relative to VSMOW (‰); mean of 2–3 injections
delta_18 O	Oxygen-18 relative to VSMOW (‰); mean of 2–3 injections
delta_2H_StDev	Standard deviation of the injections used for calculating the mean deuterium value, relative to VSMOW (‰)
delta_18O_StDev	Standard deviation of the injections used for calculating the mean oxygen-18 value, relative to VSMOW (‰)
lc_excess	Line-conditioned excess (‰) after^28^, using the LMWL equation δ^2^H = 12.9 + 8.2∙δ^18^O
rel_humidity	Daily average relative humidity (%) measured at the Erlenbach meteorological station
air_temperature	Daily average air temperature (°C) measured at the Erlenbach meteorological station
snow_depth	Daily average snow depth (cm) measured at the Erlenbach meteorological station
isotopes_data_quality	Classifies the isotope data into three groups (1 = good, 2 = potentially compromised, 3 = unreliable) to facilitate filtering the data based on data quality. We provide class-3 isotope data for information purposes only; they should not be used for analyses.
notes_sampling	Information about sampling conditions, possible storage artifacts or anomalous measurement values
notes_other	Information about hydrometeorologic measurements
notes_snow	Information about whether precipitation sampling might have been affected by snow accumulation or melt in the collection funnel. The two possible scenarios are “snow accumulation” (i.e., no sample despite recorded precipitation because snow accumulated in the collection funnel) and “snow melt” (i.e., sample despite no recorded precipitation because accumulated snow melted).

## Technical Validation

We have used several international and commercially available standards to validate our isotope measurements and to report them relative to the VSMOW-SLAP scale of the International Atomic Energy Agency IAEA (see Sect. 2.4).

The WSL isotope analyzers were calibrated with at least two International Atomic Energy Agency (IAEA) standards (VSMOW2 and SLAP2), so that isotope measurements were comparable across laboratories and instruments. The ETHZ quality control standards were validated through Isotope Ratio Mass Spectrometry (IRMS) analysis (Delta V^TM^; Thermo Fisher Scientific Inc., Massachusetts, USA) roughly every 6–8 months, using VSMOW, SLAP and five commercially available working standards from LGR for instrument calibration. IRMS measurements were carried out following the method described in Gehre *et al*.^37^.

**Fig. 4:**
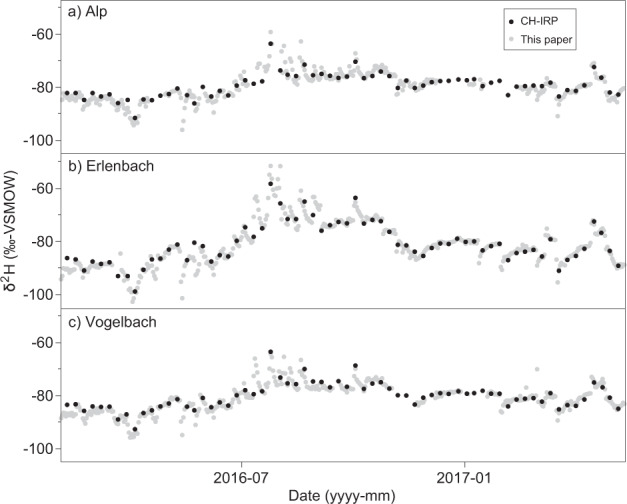
Comparison of daily (this data set, grey dots) and weekly (CH-IRP, black dots) isotope data measured in streamflow at the Alp, Erlenbach and Vogelbach catchment outlets.

In addition, we compared our daily streamwater isotope measurements against the publicly available CH-IRP data set, which contains weekly streamflow isotope measurements for the Alp, Erlenbach and Vogelbach catchments^14^. The CH-IRP streamwater samples were analyzed at the laboratory of the Chair of Hydrology at the University of Freiburg, Germany. Figure 4 shows that both data sets are generally consistent with one another. Due to the greater temporal resolution of the daily isotope measurements provided here, they can resolve short-term responses in streamflow isotopes to precipitation events that otherwise remain hidden when samples are collected weekly or monthly^13,38^.

## Supplementary information

Data Set Documentation

## Data Availability

No custom code was used to process the data described in this paper.
