# Supplementary material for: Four years of daily stable water isotope data in stream water and precipitation from three Swiss catchments
Source: Sci Data. 2022 Feb 10;9:46. doi: 10.1038/s41597-022-01148-1 (PMC8831500; doi:10.1038/s41597-022-01148-1)
Supplement: Supplementary file 1 — Data Set Documentation [file 41597_2022_1148_MOESM1_ESM.pdf]

– READ ME FIRST – Data Set Documentation

Accompanying document to:

Four years of daily stable water isotope measurements in stream water and precipitation from three Swiss catchments

Jana von Freyberg[1,2,3], Andrea Rücker[2,3], Massimiliano Zappa[2], Alessandro Schlumpf[2], Bjørn Studer[3], James W. Kirchner[2,3,4]

[1]School of Architecture, Civil and Environmental Engineering, EPFL, 1015 Lausanne, Switzerland
[2]Mountain Hydrology and Mass Movements, Swiss Federal Institute for Forest, Snow and Landscape Research (WSL), 8903 Birmensdorf, Switzerland
[3]Department of Environmental Systems Science, ETHZ, 8092 Zurich, Switzerland
[4]Department of Earth and Planetary Science, University of California, Berkeley, CA 94720, USA

Correspondence to: Jana von Freyberg (jana.vonfreyberg@epfl.ch)
James W. Kirchner (kirchner@ethz.ch)

Data archive

These data sets are made publicly available for use in education and research. The data sets are archived at https://www.doi.org/10.16904/envidat.242 (doi: 10.16904/envidat.242).

Disclaimer

These data are provided "as is", with no guarantee that they are free of errors or omissions. Those who use the data contained herein do so entirely at their own risk, and are solely responsible for determining their suitability for any particular purpose. The authors and their respective employers make no warranty, express or implied, including warranties of merchantability and fitness for a particular purpose, with respect to any information or data contained in this distribution package. The authors and their respective employers assume no legal liability for the accuracy, completeness, or usefulness of any information, apparatus, product, or process disclosed herein and do not represent that use of such information, apparatus, product or process would not infringe on privately owned rights.

Sampling and analysis procedure

For details on the sampling and analysis procedure, please refer to the main manuscript.

Column headers of the data set

The following table explains the column headers of daily stable water isotope data and hydrometeorological measurements collected at the Alptal catchment in Switzerland. The data set is provided as a single, comma-delimited .txt file.

Column Header	Explanation
date	The date that the sample was collected or the hydrometeorologic measurements were made.<br>Stream samples consist of four combined 100-ml grab samples collected at 5:40 AM, 11:40 AM, 5:40 PM, and 11:40 PM (UTC+1) on the 'date'. For precipitation samples, 'date' corresponds to the start of the sampling interval, which is from 5:40 AM of the current 'date' till 5:39 AM of the following one.<br>Ten-minute measurements of relative humidity, temperature and snow depth were averaged to daily values over the interval between 5:40 AM of the current 'date' and 5:30 AM of the following one.<br>Format: yyyy-mm-dd
catchment	Name of the study catchment
location_ID	Identifier of the sampling and/or measuring location
source	Distinguishes between precipitation or streamwater samples
waterflux_measured	Daily stream discharge (mm day^{-1}) or precipitation fluxes (mm day^{-1}) of the time interval between 5:40 AM of the current 'date' and 5:39 AM of the following one.
precipitation_interpolated	Daily, basin-average precipitation fluxes (mm day^{-1}) of the time interval between 5:40 AM of the current 'date' and 5:39 AM of the following one.
analysis_method	Method and laboratory location of the water sample analyses: L2130-*i* Picarro at WSL and ETHZ (PIC_WSL, PIC_ETHZ), Triple Isotope Water Analyzer TIWA-45EP at WSL and ETHZ (LGR_WSL, LGR_ETHZ)
sample_volume	Volume of the collected precipitation sample (ml)
delta_2H<br>delta_18O	Deuterium relative to VSMOW (‰); mean of 2-3 injections<br>Oxygen-18 relative to VSMOW (‰); mean of 2-3 injections
delta_2H_StDev	Standard deviation of the injections used for calculating the mean deuterium value, relative to VSMOW (‰)
delta_18O_StDev	Standard deviation of the injections used for calculating the mean oxygen-18 value, relative to VSMOW (‰)
lc_excess	Line-conditioned excess (‰), using the LMWL equation $\delta^{2}H=12.9+8.2\cdot\delta^{18}O$
rel_humidity	Daily average relative humidity (%) measured at the Erlenbach meteorological station
air_temperature	Daily average air temperature (°C) measured at the Erlenbach meteorological station
snow_depth	Daily average snow depth (cm) measured at the Erlenbach meteorological station
isotopes_data_quality	Classifies the isotope data into three groups (1=good, 2=potentially compromised, 3=unreliable) to facilitate filtering the data based on data quality. We provide class-3 isotope data for information purposes only; they should not be used for analyses.
notes_sampling	Information about sampling conditions, possible storage artifacts or anomalous measurement values
notes_other	Information about hydrometeorologic measurements
notes_snow	Information about whether precipitation sampling might have been affected by snow accumulation or melt in the collection funnel. The two possible scenarios are "snow accumulation" (i.e., no sample despite recorded precipitation because snow accumulated in the collection funnel) and "snow melt" (i.e., sample despite no recorded precipitation because accumulated snow melted).

Site Identifier and Catchment Area

Catchment	location_ID	Site description	Longitude (WGS84)	Latitude (WGS84)	Elevation (m above sea level)	Catchment area (km^2)
Alp	ALP_Outlet	Streamflow gauge, streamwater sampling	8.73928E	47.15080N	840	46.4
	ALP_Meteo	Meteorological station, precipitation sampling	8.75708E	47.13370N	910	
Erlenbach	ERL_Outlet	Streamflow gauge, streamwater sampling	8.70921E	47.04480N	1180	0.7
	ERL_Meteo	Meteorological station, precipitation sampling	8.71502E	47.04249N	1228	
Vogelbach	VOG_Outlet	Streamflow gauge, streamwater sampling	8.71614E	47.07621N	1050	1.6
	VOG_Precip	Rain gauge	8.71336E	47.07758N	1145	
